# Improved Prostate-Specific Membrane Antigen (PSMA) Stimulation Using a Super Additive Effect of Dutasteride and Lovastatin In Vitro

**DOI:** 10.3390/ijms241512338

**Published:** 2023-08-02

**Authors:** Aleksandar Kuzmanov, Souzan Salemi, Florian A. Schmid, Irene A. Burger, Daniel Eberli, Benedikt Kranzbühler

**Affiliations:** 1 Laboratory for Urologic Oncology and Stem Cell Therapy, Department of Urology, University Hospital Zürich, University of Zurich, 8091 Zurich, Switzerland; 2 Department of Nuclear Medicine, University Hospital Zurich, University of Zurich, 8091 Zurich, Switzerland; 3 Department of Nuclear Medicine, Baden Cantonal Hospital, 5404 Baden, Switzerland

**Keywords:** prostate-specific membrane antigen (PSMA), androgen receptor (AR), dutasteride (Duta), lovastatin (Lova), dutasteride + lovastatin (Duta + Lova) treatment

## Abstract

Prostate-specific membrane antigen (PSMA)-based imaging improved the detection of primary, recurrent and metastatic prostate cancer. However, in certain patients, a low PSMA surface expression can be a limitation for this promising diagnostic tool. Pharmacological induction of PSMA might be useful to further improve the detection rate of PSMA-based imaging. To achieve this, we tested dutasteride (Duta)—generally used for treatment of benign prostatic enlargement—and lovastatin (Lova)—a compound used to reduce blood lipid concentrations. We aimed to compare the individual effects of Duta and Lova on cell proliferation as well as PSMA expression. In addition, we tested if a combination treatment using lower concentrations of Duta and Lova can further induce PSMA expression. Our results show that a treatment with ≤1 μM Duta and ≥1 μM Lova lead to a significant upregulation of whole and cell surface PSMA expression in LNCaP, C4-2 and VCaP cells. Lower concentrations of Duta and Lova in combination (0.5 μM Duta + 0.5 μM Lova or 0.5 μM Duta + 1 μM Lova) were further capable of enhancing PSMA protein expression compared to a single compound treatment using higher concentrations in all tested cell lines (LNCaP, C4-2 and VCaP).

## 1. Introduction

Prostate-specific membrane antigen (PSMA) is a transmembrane folate-hydrolase/carboxypeptidase initially described in 1993 [1]. PSMA expression gradually increases from benign prostatic hyperplasia to adenocarcinoma of the prostate [2,3]. A high PSMA expression is primarily observed in aggressive and metastatic prostate cancer making PSMA a promising target for imaging and therapy [4]. Recently, the FDA has approved a targeted radioligand therapy using 177 Lu-PSMA-617 for the treatment of patients with PSMA-positive metastatic castration-resistant prostate cancer (mCRPC) pre-treated with androgen receptor pathway inhibitors and taxane-based chemotherapy [5].

PSMA-based imaging has significantly enhanced prostate cancer diagnostics [6]. Especially, the detection of metastases in patients with biochemical recurrence has been improved compared to other morphologic and molecular imaging modalities [7]. In addition, improved diagnostic accuracy in primary prostate cancer has been demonstrated [8,9]. Despite these improvements, the detection of significant disease is still limited in certain patients—predominantly those presenting with low PSMA expression disease at the time of recurrence and in patients with low-grade cancer at diagnosis [10]. Short-term pharmacological boosting of PSMA expression might be a valuable concept to improve prostate cancer detection using PSMA-based imaging.

Previous in vitro studies demonstrated an indirect crosslink between PSMA and androgen receptor (AR) expression [11,12]. Androgen deprivation therapy (ADT) leads to PSMA induction in primary prostate cancer tissue samples [13]. Furthermore, androgen suppression using bicalutamide, abiraterone, enzalutamide and apalutamide showed PSMA upregulation in vitro as well as in patient case reports [14,15,16,17]. In addition to the AR related regulation of PSMA, Bakht et al. showed HOXB13 transcription factor to be a direct regulator of PSMA in AR-positive and AR-negative prostate cancer cells [18]. HOXB13 knockdown led to PSMA depletion. In contrast, overexpression of HOXB13 led to PSMA induction [18].

Our previous research demonstrated that high concentrations of dutasteride (Duta) led to a significant induction of PSMA expression in LNCaP cells [19]. Duta is a well-established 5α-reductase inhibitor, with an acceptable toxicity profile, regularly used for the treatment of benign prostatic enlargement [20]. As higher concentrations of Duta were shown to increase PSMA expression, we further hypothesized that a significant induction of PSMA expression might also be achieved by using lower concentrations of Duta in combination with other approved compounds. Lovastatin (Lova) is a cholesterol lowering compound which inhibits the rate-limiting enzyme of the mevalonate pathway, 3-hydroxy-3-methylglutaryl coenzyme A (HMG-CoA) reductase [21]. It has been shown to be an AR inhibitor with a low toxicity profile that inhibits prostate cancer cell growth [22,23,24]. In our present study, we aimed to investigate the effect of Lova alone and the combination Duta + Lova on cell viability as well as PSMA and AR expression in three established prostate cancer cell lines (LNCaP, C4-2 and VCaP).

## 2. Results

### 2.1. Effect of Dutasteride and Lovastatin Single Treatment and Its Combinations on Prostate Cancer Cell Viability

**Figure 1:**
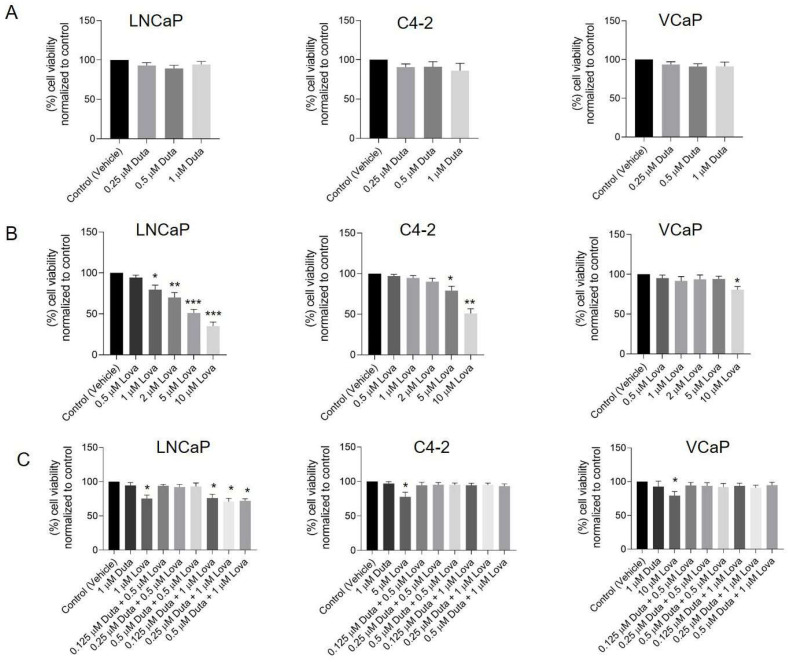
Treatment with different concentrations of Lova alone and the combination of Duta + Lova leads to reduced prostate cancer cell viability. Cell viability was assessed by CellTiter-Glo 2.0 assay. LNCaP and C4-2 cells were treated for 7 days with vehicle control (0.1% DMSO) or different concentrations of (**A**) Duta (0.25, 0.5 and 1 μM), (**B**) Lova (0.5, 1, 2, 5 and 10 μM) and (**C**) Duta + Lova combination (0.125 μM Duta + 0.5 μM Lova, 0.25 μM Duta + 0.5 μM Lova, 0.5 μM Duta + 0.5 μM Lova, 0.125 μM Duta + 1 μM Lova, 0.25 μM Duta + 1 μM Lova and 0.5 μM Duta + 1 μM Lova). VCaP cells were treated with the same conditions (Duta, Lova and Duta + Lova combination) for 14 days. The results are presented as percentage of cell viability normalized to control. Data are shown as mean with standard error of the mean (±SEM) of three independent experiments: * *p* < 0.05, ** *p* < 0.01, *** *p* < 0.001. DMSO, dimethyl sulfoxide.

Initial experiments using a CellTiter-Glo 2.0 assay were performed to analyze concentration dependent effects of Duta and Lova separately and in combination on cell viability. Cell viability was not significantly reduced when LNCaP and C4-2 cells were treated for 7 days with 0.25, 0.5 and 1 μM Duta (Figure 1A). Treating VCaP cells for both 7 (Supplementary Figure S1A) and 14 days (Figure 1A) using the same Duta concentrations did not influence cell viability either.

A treatment of LNCaP cells for 7 days with increasing Lova concentrations led to a significant reduction of cell viability compared to vehicle control (20% for 1 μM Lova, *p* < 0.05, 30% for 2 μM Lova, *p* < 0.01, 49% for 5 μM Lova, *p* < 0.001 and 65% for 10 μM Lova, *p* < 0.001; Figure 1B). C4-2 cells showed a similar viability pattern. However, cell viability was only significantly reduced using 5 μM (21%, *p* < 0.05) and 10 μM (49%, *p* < 0.01) Lova (Figure 1B). Cell viability of VCaP cells was not affected by any of the concentrations tested upon treatment for 7 days (Supplementary Figure S1B). In contrast, prolonging the treatment of VCaP cells for 14 days showed a significant decrease in cell viability, specifically by 20% when high concentrations of Lova (10 μM, *p* < 0.05: Figure 1B) were used.

Further experiments explored the effect of different Duta + Lova combinations using lower single compound concentrations on cell viability. The concentrations tested were as follows: 0.125 μM Duta + 0.5 μM Lova, 0.25 μM Duta + 0.5 μM Lova, 0.5 μM Duta + 0.5 μM Lova, 0.125 μM Duta + 1 μM Lova, 0.25 μM Duta + 1 μM Lova and 0.5 μM Duta + 1 μM Lova. A treatment of LNCaP cells for 7 days with 0.125 μM Duta + 1 μM Lova (24%), 0.25 μM Duta + 1 μM Lova (30%) and 0.5 μM Duta + 1 μM Lova (39%) led to a significant decrease (*p* < 0.01) in cell viability compared to the control, but not compared to cells treated with 1 μM Duta or 1 μM Lova alone (Figure 1C). Interestingly, in C4-2 cells the combination Duta + Lova did not alter cell viability compared to vehicle control (Figure 1C). Similar results were observed in VCaP cells. Both 7 (Supplementary Figure S1C) and 14 days (Figure 1C) treatment of Duta + Lova combined treatment had no effect on VCaP cell viability.

### 2.2. Total PSMA and AR Protein Expression following Pharmacological Stimulation

We then investigated the impact of Duta and Lova separately and in combination on PSMA and AR expression using protein simple immunoblotting.

### 2.3. Impact of Dutasteride Single Treatment

**Figure 2:**
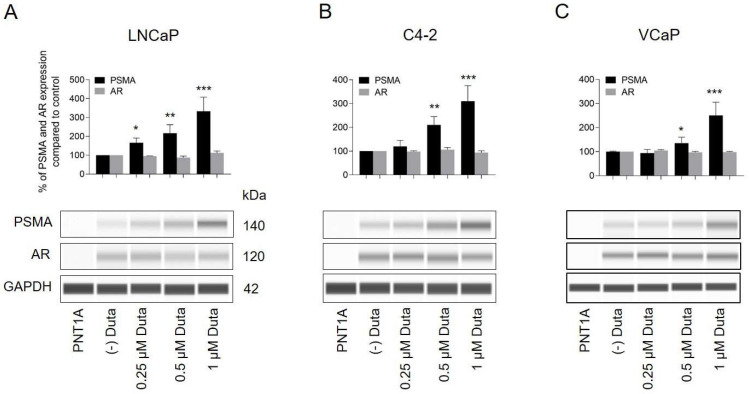
Treatment of prostate cancer cells with different concentrations of Duta leads to increased total PSMA expression. Whole PSMA protein expression analyzed by protein simple immunoblotting. (**A**) LNCaP and (**B**) C4-2 cells were treated for 7 days with different concentrations of Duta (0.25, 0.5 and 1 μM). (**C**) VCaP cells were treated for 14 days with the same Duta concentrations. PSMA and AR expression is presented as the percentage of PSMA and AR expression compared to vehicle control. Data are shown as mean with standard error of the mean (±SEM) of three independent experiments. AR, androgen receptor; PSMA, prostate-specific membrane antigen. * *p* < 0.05, ** *p* < 0.01, *** *p* < 0.001.

Treatment of LNCaP cells with 0.25 μM (166 ± 25%, *p* < 0.05), 0.5 μM (217 ± 44%, *p* < 0.01) and 1 μM Duta (333 ± 75%, *p* < 0.001) led to a significant increase in total PSMA expression compared to vehicle control (Figure 2A). In C4-2 cells, a treatment using 0.5 μM (210 ± 35%, *p* < 0.01) and 1 μM Duta (310 ± 62%, *p* < 0.001) significantly induced total PSMA expression compared to vehicle control (Figure 2B). Total PSMA expression in VCaP cells following Duta treatment for 14 days was comparable to the pattern observed in C4-2 cells treated for 7 days (Figure 2C). Treatment of VCaP cells for 7 days did not significantly alter total PSMA protein expression (Supplementary Figure S2A). In contrast to PSMA, AR expression showed no significant change in LNCaP, C4-2 and VCaP cells following treatment with different concentrations of Duta (Figure 2 and Supplementary Figure S2A).

### 2.4. Impact of Lovastatin Single Treatment

**Figure 3:**
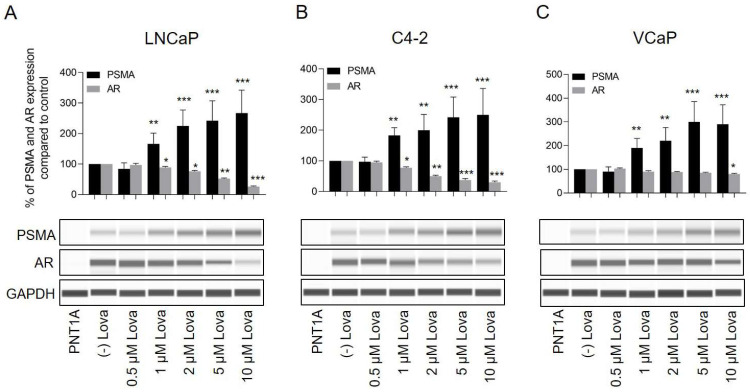
Treatment of prostate cancer cells with different concentrations of Lova leads to increases in total PSMA expression and reduced AR expression. Whole PSMA protein expression analyzed using protein simple immunoblotting. (**A**) LNCaP and (**B**) C4-2 cells were treated for 7 days with different concentrations of Lova (0.5, 1, 2, 5 and 10 μM). (**C**) VCaP cells were treated for 14 days with the same Lova concentrations. PSMA and AR expressions are presented as the percentage of PSMA and AR expression compared to vehicle control. Data are shown as mean with standard error of the mean (±SEM) of three independent experiments. * *p* < 0.05, ** *p* < 0.01, *** *p* < 0.001.

Furthermore, total PSMA expression was significantly increased following treatment of LNCaP cells with 1 μM (167 ± 35%, *p* < 0.01), 2 μM (225 ± 52%, *p* < 0.001), 5 μM (242 ± 66%, *p* < 0.001) and 10 μM (267 ± 74%, *p* < 0.001) of Lova compared to control (Figure 3A). Similar results were observed in C4-2 cells (1 μM; 183 ± 25%, 2 μM; 200 ± 51%, 5 μM; 243 ± 64%; and 10 μM; 250 ± 86%: Figure 3B). Treatment of VCaP cells with Lova for 7 days did not significantly change PSMA protein expression (Supplementary Figure S2B). Therefore, VCaP cells were treated for up to 14 days to check whether PSMA induction might occur at a later time point. Interestingly, treatment of VCaP cells for 14 days led to a similar PSMA induction compared to a treatment of LNCaP and C4-2 cells for 7 days (Figure 3C).

In addition to PSMA, AR expression was assessed. LNCaP cells treated for 7 days with 1 μM (88 ± 3%, *p* < 0.05), 2 μM (76 ± 4%, *p* < 0.05), 5 μM (52 ± 3%, *p* < 0.01) and 10 μM (26 ± 2%, *p* < 0.001) Lova demonstrated a significant inhibition of AR expression (Figure 3A). Similar results were observed in C4-2 cells (1 μM: 77 ± 3%, *p* < 0.05; 2 μM: 50 ± 3%, *p* < 0.01; 5 μM: 37 ± 5%, *p* < 0.001; and 10 μM: 30 ± 4%, *p* < 0.001). Comparable to PSMA expression patterns, no AR alteration was observed in VCaP cells treated for 7 days with Lova (Supplementary Figure S2B). However, a treatment of VCaP cells for 14 days led to a significant reduction (*p* < 0.05) of AR expression in cells treated with 10 μM (80 ± 3%) Lova (Figure 3C).

### 2.5. Combination Treatment of Dutasteride + Lovastatin

**Figure 4:**
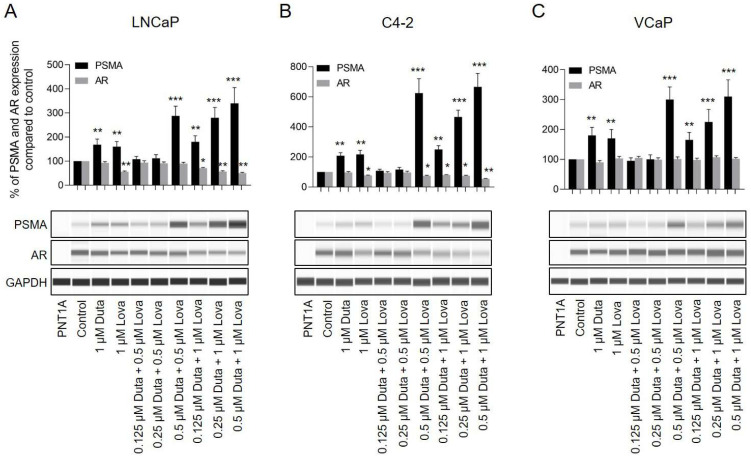
Treatment of prostate cancer cells with different concentrations of Duta + Lova leads to increased total PSMA expression and reduced AR expression. Whole PSMA protein expression analyzed by protein simple immunoblotting. (**A**) LNCaP and (**B**) C4-2 cells were treated for 7 days with different concentrations of Duta + Lova combination (0.125 μM Duta + 0.5 μM Lova, 0.25 μM Duta + 0.5 μM Lova, 0.5 μM Duta + 0.5 μM Lova, 0.125 μM Duta + 1 μM Lova, 0.25 μM Duta + 1 μM Lova and 0.5 μM Duta + 1 μM Lova). (**C**) VCaP cells were treated for 14 days with the same Duta + Lova concentrations. PSMA and AR expressions are presented as the percentage of PSMA and AR expression compared to vehicle control. Data are shown as mean with standard error of the mean (±SEM) of three independent experiments. * *p* < 0.05, ** *p* < 0.01, *** *p* < 0.001.

A combination treatment of LNCaP cells using 0.5 μM Duta + 0.5 μM Lova (288 ± 42%, *p* < 0.001), 0.25 μM Duta + 1 μM Lova (280 ± 44%, *p* < 0.001) and 0.5 μM Duta + 1 μM Lova (340 ± 65%, *p* < 0.001) led to a further significant increase in total PSMA expression compared to single compound treatment using 1 μM Duta (168 ± 24%, *p* < 0.01) or 1 μM Lova (160 ± 22%, *p* < 0.01) (Figure 4A). A comparable PSMA expression pattern was observed in 7 days treated C4-2 cells and in 14 days treated VCaP cells (Figure 4B,C). VCaP cells treated for 7 days showed no alteration in PSMA protein expression (Supplementary Figure S2C). In LNCaP cells, total AR expression did not show any additional decrease compared to single compound treatment using 1 μM Duta or 1 μM Lova (Figure 4A). In C4-2 cells, AR expression was further reduced using 0.5 μM Duta + 1 μM Lova (52 ± 2%, *p* < 0.01) compared to treatment with 1 μM Lova (77 ± 3%, *p* < 0.05) (Figure 4B). In VCaP cells, AR expression was unaffected by both a 7 and 14 days combination treatment using Duta and Lova (Figure 4C and Supplementary Figure S2C).

### 2.6. PSMA and AR Surface Expression following Stimulation with Dutasteride and Lovastatin

**Figure 5:**
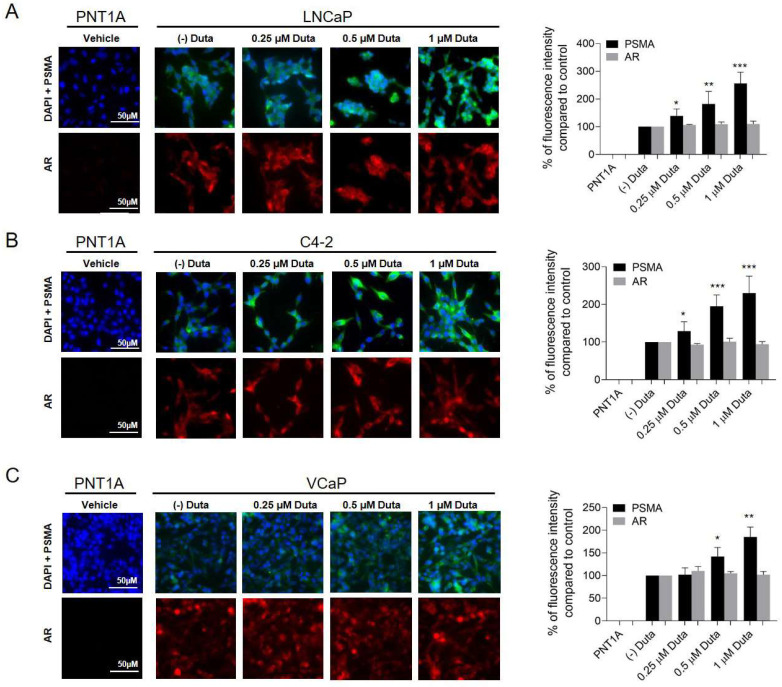
PSMA surface expression is induced in prostate cancer cells following stimulation with Duta. Visualization of surface PSMA and AR expression using immunocytochemistry after Duta treatment. Images were taken with Leica Thunder inverted microscope. LNCaP cells (**A**) and C4-2 cells (**B**) were cultured on chamber slides and incubated for 7 days with vehicle control (0.1% DMSO) or different concentrations of Duta (0.25, 0.5 and 1 μM). (**C**) VCaP cells were cultured for 14 days with the same Duta concentrations. PSMA and AR cell surface expressions are presented as the percentage of fluorescence intensity compared to control. * *p* < 0.05, ** *p* < 0.01, *** *p* < 0.001. Scale bars indicate 50 μM.

The visualization of cell surface PSMA and AR expression in prostate cancer cells was performed by immunocytochemistry. LNCaP cells treated for 7 days with 0.25 μM (139 ± 25%, *p* < 0.05), 0.5 μM (182 ± 45%, *p* < 0.01) and 1 μM (256 ± 40% *p* < 0.001) Duta showed a significant increase in cell surface PSMA expression compared to the vehicle control (Figure 5A). Similar results were observed in C4-2 cells (Figure 5B). In VCaP cells, only treatments with 0.5 μM (143 ± 21%, *p* < 0.05) and 1 μM (185 ± 22% *p* < 0.01) Duta for 14 days showed a significant increase in cell surface PSMA expression (Figure 5C). However, AR expression was not influenced in LNCaP, C4-2 and VCaP cells when different concentrations of Duta were applied (Figure 5).

**Figure 6:**
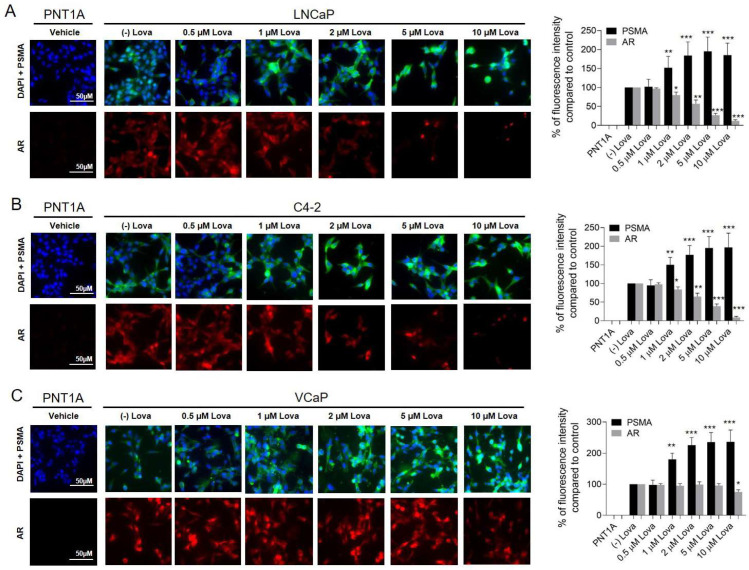
Treatment of prostate cancer cells with different concentrations of Lova leads to increased cell surface PSMA expression and reduced AR expression. Visualization of surface PSMA and AR expression using immunocytochemistry after Lova treatment. LNCaP cells (**A**) and C4-2 cells (**B**) were cultured on chamber slides and incubated for 7 days with vehicle control (0.1% DMSO) or different concentrations of Lova (0.5, 1, 2, 5 and 10 μM). (**C**) VCaP cells were cultured for 14 days with the same Lova concentrations. PSMA and AR cell surface expressions are presented as the percentage of fluorescence intensity compared to control. * *p* < 0.05, ** *p* < 0.01, *** *p* < 0.001. Scale bars indicate 50 μM.

Treatment of LNCaP cells with 1 μM (152 ± 30%, *p* < 0.01), 2 μM (184 ± 36%, *p* < 0.001), 5 μM (195 ± 38%, *p* < 0.001) and 10 μM (185 ± 32%, *p* < 0.001) Lova showed a significant increase in cell surface PSMA expression compared to the control (Figure 6A). These results were confirmed in C4-2 cells treated for 7 days and VCaP cells treated for 14 days (Figure 5B,C). AR expression in LNCaP cells treated with 1 μM (80 ± 8%, *p* < 0.05), 2 μM (57 ± 10%, *p* < 0.01), 5 μM (27 ± 5%, *p* < 0.001) and 10 μM (13 ± 3%, *p* < 0.001) Lova was significantly reduced compared to the vehicle control (Figure 6A). Comparable results were observed in C4-2 cells (Figure 6B). In VCaP cells, only a treatment with 10 μM (75 ± 7%, *p* < 0.05) Lova led to a significant reduction in AR expression (Figure 6C).

**Figure 7:**
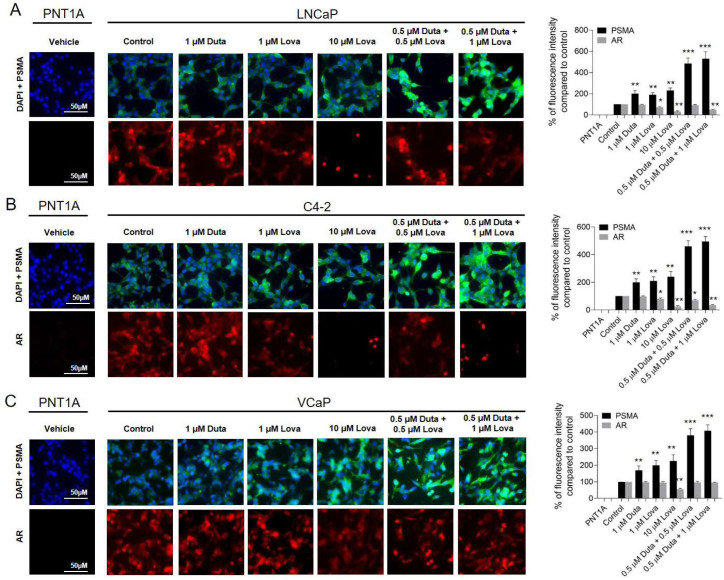
Treatment of prostate cancer cells with different concentrations of Duta + Lova leads to increases cell surface PSMA expression and reduced AR expression. Visualization of surface PSMA and AR expression using immunocytochemistry after combined Duta/Lova treatment. LNCaP cells (**A**) and C4-2 cells (**B**) were cultured on chamber slides and incubated for 7 days with vehicle control (0.1% DMSO) or different concentrations of Duta + Lova (0.125 μM Duta + 0.5 μM Lova, 0.25 μM Duta + 0.5 μM Lova, 0.5 μM Duta + 0.5 μM Lova, 0.125 μM Duta + 1 μM Lova, 0.25 μM Duta + 1 μM Lova and 0.5 μM Duta + 1 μM Lova). (**C**) VCaP cells were cultured for 14 days with the same Duta + Lova concentrations. PSMA and AR cell surface expressions are presented as the percentage of fluorescence intensity compared to control. * *p* < 0.05, ** *p* < 0.01, *** *p* < 0.001. Scale bars indicate 50 μM.

A combination treatment of LNCaP cells with 0.5 μM Duta + 0.5 μM Lova (485 ± 52%, *p* < 0.001), and 0.5 μM Duta + 1 μM Lova (530 ± 66%, *p* < 0.001) led to a further significant increase in cell surface PSMA expression compared to single compound treatments with 1 μM Duta (199 ± 29%, *p* < 0.01) or 1 μM Lova (190 ± 20%, *p* < 0.01) (Figure 7A). These results were also confirmed in C4-2 cells and VCaP cells treated for 14 days (Figure 7B,C). In LNCaP cells, AR expression was significantly reduced using 0.5 μM Duta + 1 μM Lova (48 ± 5%, *p* < 0.01) compared to treatments with 1 μM Duta, 1 μM Lova or 0.5 μM Duta + 0.5 μM Lova (Figure 7A). These results were observed in C4-2 cells as well (Figure 7B). In VCaP cells, AR expression did not show any additional decrease compared to single compound treatments using 1 μM Duta or 1 μM Lova (Figure 7C).

## 3. Discussion

In this study, we were able to show for the first time that Duta and Lova significantly induce PSMA overexpression in different well-established prostate cancer cell lines (LNCaP, C4-2 and VCaP). In addition, our investigations revealed that a combination treatment using lower concentrations of Duta and Lova further enhances total and cell surface PSMA protein expression compared to single compound treatments using higher concentrations, respectively. These results urge us to further analyze combination treatments as potential inducers of PSMA surface expression prior to PSMA-based imaging.

Despite implementation of PSMA-based imaging and therapy into clinical practice, specific functions, and regulations of PSMA are yet to be fully understood. Recently published work suggested that the enzymatic function of PSMA is cleaving glutamate, thereby activating the glutamate-driven phosphoinositide 3-kinase and subsequently the mTOR pathway, leading to tumor cell survival and growth [25,26]. In addition, regulation of PSMA has been demonstrated to be androgen-dependent [11,13,27,28]. Meller et al. found an increased PSMA expression following short-term treatment of prostate cancer cells using second generation ADT with abiraterone [16]. Another study suggested a time-dependent effect of AR inhibition using enzalutamide on PSMA expression in vitro [17]. These results led to a first promising patient report of PSMA upregulation detected by ^68^Ga-PSMA-11 PET/MRI following 4 weeks of treatment with bicalutamide and a single injection of leuprolide acetate [14]. However, first (bicalutamide) and second (abiraterone, enzalutamide) generation androgen deprivation therapies are associated with significant side effects in patients and high costs. Furthermore, the first human data suggested that an increased uptake of ^68^Ga-PSMA-11 following androgen inhibition is only seen in patients presenting with mCRPC and not in patients with hormone-sensitive PCa [29]. Therefore, these compounds might not be ideal candidates for pharmacological induction of PSMA expression in patients with primary prostate cancer or early biochemical recurrence. However, different PSMA inducing compounds might overcome this drawback. Sufficient PSMA induction might improve therapy effects of ^177^Lu-PSMA-617 in patients with PSMA-positive mCRPC [5], and furthermore, it may enhance detection rates of primary hormone-sensitive PCa.

Duta is an alternative compound with an acceptable toxicity profile that is widely used for the treatment of lower urinary tract symptoms due to benign prostatic enlargement. Thus, Duta could be implemented with ease into clinics as a possible PSMA expression enhancer prior to PSMA-based imaging [20]. Having in mind that the highest serum concentrations observed in men reached above 1 µM given an oral dose of 5 mg dutasteride daily [30], only Duta concentrations of up to 1 µM were used in our current study.

Lazier et al. revealed that high concentrations of Duta promote loss of AR proteins and increase cell death in LNCaP cells [31]. Conflicting results were published by Chhipa et al., showing that mutations in the ligand-binding domain of the AR do not significantly influence the inhibitory effect of Duta on the AR [32]. Our results support the data from Chhipa et al. showing that treatment of LNCaP, C4-2 and VCaP cells with 0.25 μM, 0.5 μM and 1 μM Duta does not affect AR expression and cell viability.

Since prostate cancer cell growth is AR dependent [23,33,34] we consider the lack of AR expression changes responsible for the absence of cell viability variations following treatment with dutasteride. In addition, we could demonstrate that a 7-day treatment using 0.25 μM, 0.5 μM and 1 μM dutasteride increased whole and cell surface PSMA protein expression in LNCaP and C4-2 cells. PSMA expression was not altered in VCaP cells treated for 7 days using the same Duta concentrations. However, a 14-day treatment of VCaP cells confirmed the results observed when LNCaP and C4-2 cells were treated for 7 days. We hypothesize that the discrepancy at day 7 post Duta treatment in VCaP cells is explained by the fact that these cells have a slower metabolism and growth rate compared to LNCaP and C4-2 cells [35]. The current results confirm our previous findings in LNCaP cells and expand our knowledge on the effects of Duta on PSMA expression in two additional prostate cancer cell lines (C4-2 and VCaP) [36]. The lack of changes in AR expression following Duta treatment suggests that the observed PSMA induction is AR independent. Moreover, it implies that other pathways might be involved in PSMA regulation. Integrin β1 and PAK (p21-activated kinase)-1 have been indicated in the activation of PSMA. PAK-1 interferes with the interaction between the PSMA cytoplasmic tail region and anchor protein filamin A to reduce the activity of PSMA, while the inhibition of Integrin β1 activity can also reduce the activity of PSMA [37]. In addition, Perico et al. have demonstrated that PSMA activity depends on the assembly of a macromolecular complex including filamin A, beta1 integrin, p130CAS, c-Src and EGFR in LNCaP and PC3 cells [25]. In addition, Bakht et al. showed HOXB13 transcription factor to be a direct regulator of PSMA in AR-positive and AR-negative prostate cancer cells [18]. A stable knock-out of HOXB13 by using CRISPR/Cas 9 led to a reduced PSMA expression, an increased tumor weight and a reduced metastatic potential. In contrary, overexpression of HOXB13 in prostate cancer cells led to an increase in PSMA expression levels [18]. Therefore, we speculate that the induction of PSMA that we observe in prostate cancer cells upon Duta treatment might be HOXB13 related and AR independent. Further analyses are needed to evaluate this hypothesis.

Lova is another compound with a low toxicity profile that is commonly used to reduce blood lipid concentrations in patients with hypercholesterolemia [23,38]. Several in vitro studies demonstrated that Lova has a significant inhibitory effect on cell viability in a variety of solid cancers (breast, liver, cervical, lung and colon cancer). Lova inhibits cell proliferation and regulates cancer cell signaling pathways, thereby inducing apoptosis and cell cycle arrest [38]. In our study, we focused on the use of clinically achievable concentrations below 10 μM [23]. Here, we report for the first time that a 7-day treatment with 1 μM Lova or higher concentrations (2, 5 and 10 μM) significantly induces whole and cell surface PSMA protein expression in LNCaP and C4-2 cells. A similar PSMA behavior was observed in VCaP cells treated for up to 14 days. The mechanism leading to PSMA induction upon Lova treatment remains largely unknown. In contrary to Duta, Lova treatment of LNCaP, C4-2 (7 days) and VCaP (14 days) cells led to a significant AR inhibition. Similar results were observed by Yang et al. [23]. They reported a significant inhibition of AR expression following treatment of LNCaP cells for 72 h with ≥ 2 μM Lova. In VCaP cells, Yang et al. reported no effects on AR. This might be explained by the short treatment window of only 72 h in which the cells were exposed to Lova. The same study demonstrated that impairment of Akt activation results in reduced AR signaling associated with the inhibition of cell proliferation and the induction of apoptosis. Others reported a reduced androgen receptor-mediated signaling through the regulation of CAV1 expression in a castration-resistant prostate by using simvastatin [39]. In addition, the relation between Lova and the newly discovered PSMA-direct regulator HOXB13 on PSMA regulation remains to be explored. As for Duta, additional functional studies are needed to better understand how Lova induces PSMA expression and to explore its role in promoting AR inhibition and cell viability.

Having in mind that a single compound treatment using Duta or Lova significantly induces PSMA expression in different prostate cancer cells, we further explored the following question: is a combination treatment using lower concentrations of Duta and Lova capable of further increasing PSMA upregulation compared to a single compound treatment? Additional experiments confirmed that a combination of Duta + Lova in lower concentrations (0.5 μM Duta + 0.5 μM Lova and 0.5 μM Duta + 1 μM Lova) further enhances PSMA protein expression compared to single compound treatments using higher concentrations of Duta and Lova. Interestingly, we did not observe any alteration in AR protein expression when a Duta + Lova combination was used. These promising results motivate us to further explore the possibility of short-term PSMA induction. Additional in vitro and in vivo studies are needed to better understand PSMA regulation and associated pathways.

## 4. Materials and Methods

### 4.1. Cell Culture

Prostate cancer cells (LNCaP and VCaP) were purchased from American Type Culture Collection (ATCC, Manassas, VA, USA). C4-2 cells were a generous gift from Prof. Dr. med. George N. Thalmann (University Hospital Bern, Bern, Switzerland). Both LNCaP and C4-2 cell lines were cultured in RPMI Medium 1640 (1×) [+] L-Glutamine (Gibco, Reinach, Switzerland) supplemented with 10% FBS and 1% penicillin/streptomycin (P/S). VCaP cells are derived from a vertebral metastatic prostate cancer lesion [24] and were grown in DMEM (BioConcept) High Glucose (4.5 g/L) with stable glutamine and sodium pyruvate, supplemented with 10% FBS and 1% penicillin/streptomycin (P/S). PNT1A epithelial cells were a generous gift from Pirkko Härkönen (Institute of Biomedicine, University of Turku, Finnland). As PSMA/AR are not expressed in PNT1A cells, these cells were used as negative controls when PSMA status was explored. All cells were incubated at 37 °C with 5% CO_2_. Cells were not used for longer than 10 passages. Medium was changed twice a week.

### 4.2. Pharmacological Compounds

LNCaP and C4-2 cells were treated for up to 7 days with 0.25, 0.5 and 1 μM Duta; 0.5, 1, 2, 5 and 10 μM Lova or a combination of both: 0.125 μM Duta + 0.5 μM Lova, 0.25 μM Duta + 0.5 μM Lova, 0.5 μM Duta + 0.5 μM Lova, 0.125 μM Duta + 1 μM Lova, 0.25 μM Duta + 1 μM Lova and 0.5 μM Duta + 1 μM Lova (all purchased from Selleckchem, Luzern, Switzerland) according to the experimental conditions. VCaP cells were treated for 14 days with the same combination of compounds. The vehicle used was 0.1% dimethyl sulfoxide (DMSO). The medium containing compounds were usually changed on the third day after initial treatment. All experiments were performed in triplicate.

### 4.3. Cell Viability Assays

Cells were plated at a density of 8 × 10^4^ cells in 96-well plate (Corning Incorporated) and cultured overnight. The next day, cells were treated with different concentrations of Duta and Lova. LNCaP and C4-2 cells were incubated at 37 °C for 7 days. VCaP cells were incubated at 37 °C for 7 and 14 days. Viability was assessed on day 7 and 14 using CellTiter-Glo 2.0 (Promega) and Cytation 5 Imaging reader at 560 nm emission (BioTek). Percentage of viable cells normalized to control was determined.

### 4.4. Immunocytochemistry

Cells were seeded on chamber slides (LabTek, ThermoFisher SCIENTIFIC, Lausanne, Switzerland) in growth medium for 1 day. The next day, cells were treated for 7 days, as mentioned above. The indirect immunostainings for cells were performed at 37 °C with overnight incubation using the primary antibodies anti-PSMA/FOLH1 (clone 460420, 1:100, R&D Systems, Zug, Switzerland) and AR (clone D6F11, 1:500, CellSignaling, Leiden, The Netherlands). The slides were incubated with secondary antibodies: goat anti-mouse FITC (1:500, Vector Laboratories) or Cy3-conjugated goat anti-rabbit antibody (Sigma, 1:1000) at room temperature for 1 h. After counter-staining with DAPI (4′, 6-diamidino-2-phenylindole, Sigma, 1:200), the slides were analyzed by Thunder imaging system (Leica DMi_8_ Thunder inverted microscope, Mannheim, Germany). Fluorescence intensity was measured using Image J (1.4, NIH, USA).

### 4.5. Protein Simple WES Immunoblotting

After 7 days of culturing, cells were washed with cold PBS supplemented with a protease inhibitor cocktail (Sigma-Aldrich, Buchs, Switzerland) and lysed with modified lysis buffer. Total protein was measured with the BCA Protein Assay Kit (ThermoFisher SCIENTIFIC, Lausanne, Switzerland). Protein at a concentration of 1 mg/mL was used for the WES sample preparation using the 12–230 kDa cartridge kit. Proteins were separated in WES with a capillary cartridge according to the manufacturer’s protocols (Protein Simple WES, Wiesbaden-Nordenstadt, Germany). Primary antibodies were mouse anti-PSMA/FOLH1 (R&D Systems, 4:100) and rabbit anti-AR (CellSignaling, 1:100). Mouse anti-GAPDH (Novus Biologicals Europe, 1:100) served as internal control.

### 4.6. Statistical Analysis

Data analysis was performed using GraphPad Prism (GraphPad Software, Inc., La Jolla, CA, USA, version 7). Mann–Whitney nonparametric *t*-test was performed to determine statistical significance. *p*-values < 0.05 were considered significant. All data presented are expressed as means with corresponding standard error of the mean (±SEM).

## 5. Conclusions

In our study, we showed a significant induction of total and surface PSMA expression upon treatment of different well-established prostate cancer cell lines with Lova. In addition, we demonstrated that a combination treatment using lower concentrations of Duta and Lova, at clinically established doses, further enhances PSMA expression compared to a single compound treatment.

## Data Availability

The datasets generated and analyzed during the current study are not publicly available. However, they are available from the corresponding author upon reasonable request.

## Supplementary Materials

The following supporting information can be downloaded at: https://www.mdpi.com/article/10.3390/ijms241512338/s1.
