# Supplementary material for: Improved Prostate-Specific Membrane Antigen (PSMA) Stimulation Using a Super Additive Effect of Dutasteride and Lovastatin In Vitro
Source: Int J Mol Sci. 2023 Aug 2;24(15):12338. doi: 10.3390/ijms241512338 (PMC10419009; doi:10.3390/ijms241512338)
Supplement: Supplementary file 1 [file ijms-24-12338-s001.zip › ijms-2520741-supplementary.pdf]

Supplementary figures

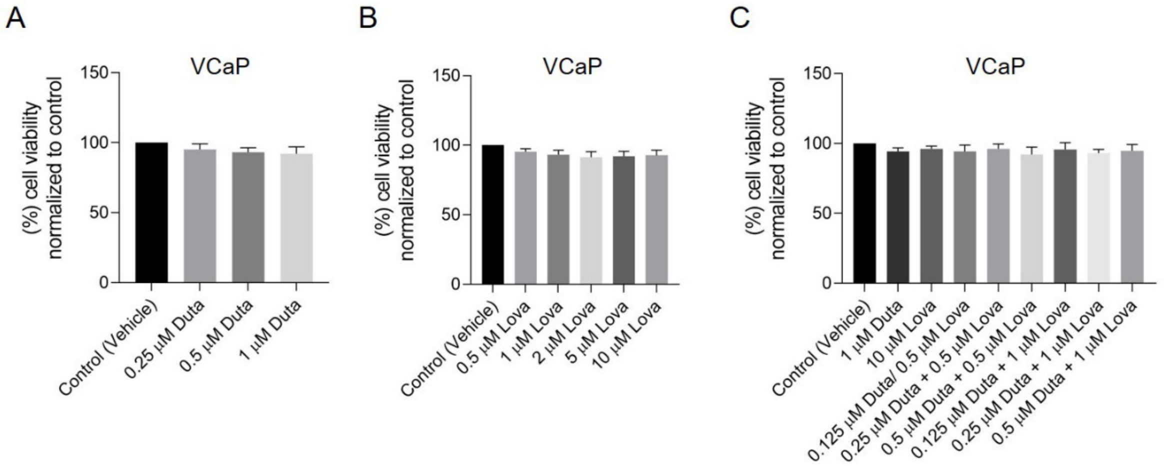

Supplementary figure S1. Cell proliferation was assessed by CellTiter-Glo 2.0 assay. VCaP cells were treated for 7 days with vehicle control (0.1% DMSO) or different concentrations of (A) Duta (0.25, 0.5 and 1 µM), (B) Lova (0.5, 1, 2, 5 and 10 µM) and (C) Duta + Lova combination (0.125 µM Duta + 0.5 µM Lova, 0.25 µM Duta + 0.5 µM Lova, 0.5 µM Duta + 0.5 µM Lova, 0.125 µM Duta + 1 µM Lova, 0.25 µM Duta + 1 µM Lova and 0.5 µM Duta + 1 µM Lova). The results are presented as percentage of cell viability normalized to control. Data is shown as mean with standard error of the mean (± SEM) of three independent experiments. DMSO, dimethyl sulfoxide.

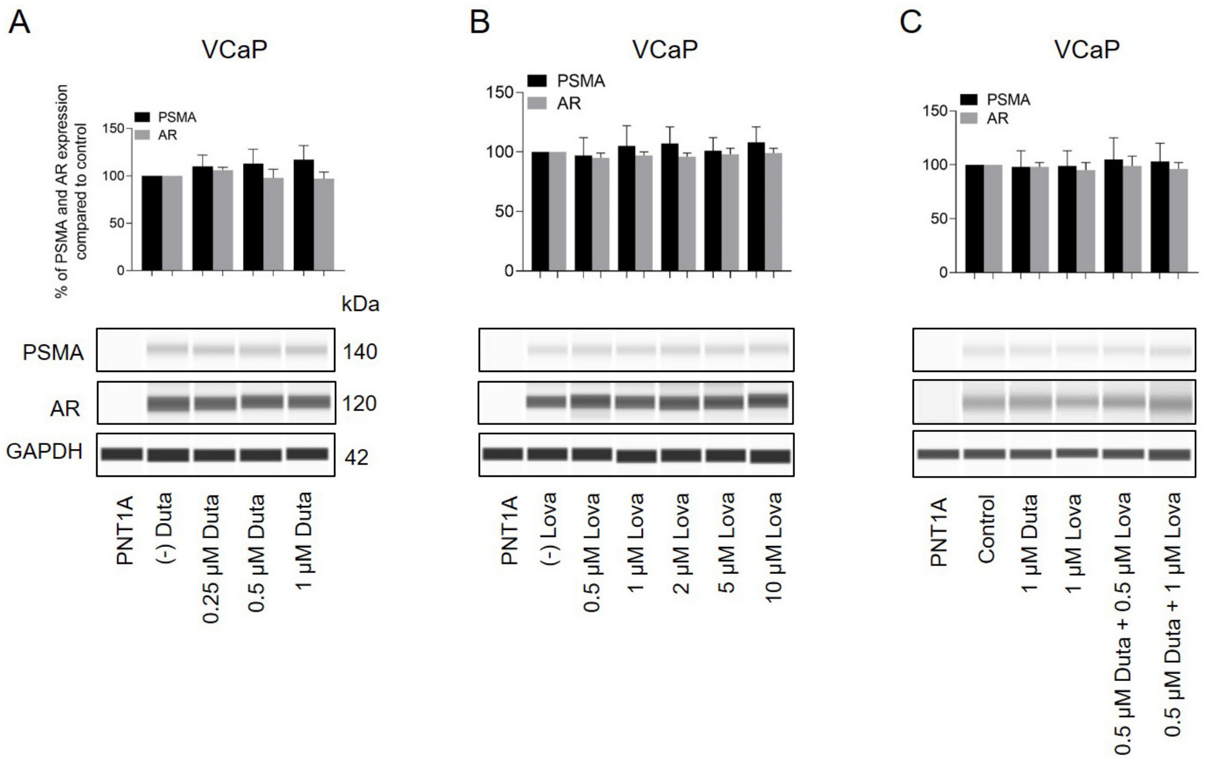

Supplementary Figure S2. Whole PSMA protein expression analyzed by protein simple immunoblotting. VCaP cells were treated for 7 days with different concentrations of (A) Duta (0.25, 0.5 and 1 µM), (B) Lova (0.5, 1, 2, 5 and 10 µM) and (C) Duta + Lova combination (0.5 µM Duta + 0.5 µM Lova and 0.5 µM Duta + 1 µM Lova). Data is shown as mean with standard error of the mean (± SEM) of three independent experiments. AR, androgen receptor; PSMA, prostate specific membrane antigen.
